# The effect of propolis extract as a valuable natural additive on the quality characteristics of toast bread

**DOI:** 10.1002/fsn3.3500

**Published:** 2023-06-14

**Authors:** Saba Hosseini Khabbazi, Samar Mansouripour, Solmaz Saremnezhad

**Affiliations:** ^1^ Department of Food Science and Technology, Faculty of Pharmacy, Tehran Medical Sciences Islamic Azad University Tehran Iran

**Keywords:** antioxidant activity, natural preservative, phenolic compounds, propolis, toast bread

## Abstract

This study aimed to evaluate the effect of ethanolic propolis extract (EPE) as a natural antimicrobial and antioxidant agent on the physicochemical, microbial, and sensory characteristics of toast bread, as well as phenol content and antioxidant activity. In this regard, 0.1, 0.3, and 0.5% of EPE were used in the bread doughs and the quality characteristics of the breads were assessed. The bread with 0.5% EPE showed the highest phenolic content (24.02 mgGAE/g.d.m) and antioxidant activity (59.03%). These amounts were 12.96 mgGAE/g and 16.45% higher than those of the control (without EPE), respectively (*p* < .05). The hardness, fracturability, and chewiness of the bread samples were influenced by the levels of EPE on the third and fifth days of storage. EPE decreased the L* and a* of bread samples, but an increasing trend was observed in the b*, chroma, and browning index by elevating the levels of propolis. Propolis extract showed an inhibitory effect on mold growth in samples. The bread with 0.5% of EPE had the lowest mold count after 5 days of storage which was not significant compared to the first day. There was no significant difference in sensory evaluation between the overall acceptance of bread samples. Therefore, EPE has the potential to be used as a natural additive with antimicrobial and antioxidant characteristics in toast bread.

## Graphical abstract

Ethanolic propolis extract was effective in the inhibition of mold growth of toast bread during storage and improved the antioxidant characteristics. The bread with 0.5 % EPE showed the highest phenolic content and antioxidant activity, as well as the lowest mold count after 5 days of storage. The overall sensory acceptance of bread samples was acceptable. Therefore, EPE can be used as a valuable natural additive with antimicrobial and antioxidant functions in toast bread.

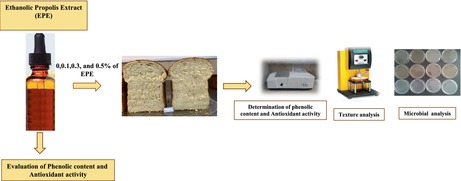

## 1 INTRODUCTION

Bread is a staple food consumed daily in many countries of the world and is a valuable source of nutrients (Axel et al., 2017; Mikulec et al., 2019). Toast bread is a leavened bread with a soft texture that is usually consumed for breakfast after being sliced (Mohammadi Golchin et al., 2021). Chemical preservatives are commonly used to increase shelf life and prevent mold growth in bread. The concentration of these additives has legal limits (Axel et al., 2017) and excess intake is detrimental to the consumer's health (Santos et al., 2020; Yang et al., 2017). Since there is a demand from health‐conscious consumers for products with natural ingredients and without chemicals, it seems necessary to utilize natural additives in bread formulation.

Propolis is a valuable compound made by honeybees after collecting resinous substances from different parts of plants and mixing them with their wax and saliva enzymes. Honeybees use propolis to fill the cracks inside the hive and also as a protective barrier against pathogens (Anjum et al., 2019; Irigoiti et al., 2021; Pobiega et al., 2019; Shehata et al., 2020; Silva et al., 2012). There are various compounds such as polyphenols, acids, terpenes, aromatic acids, resins, waxes, essential oils, other organic compounds, and minerals in propolis (Ibrahim & Alqurashi, 2022; Irigoiti et al., 2021; Pobiega et al., 2019; Przybyłek & Karpiński, 2019). Its chemical composition is different depending on the type of plants and geographical regions (Przybyłek & Karpiński, 2019; Shehata et al., 2020). The aqueous and alcoholic extracts of propolis have shown antibacterial, antifungal, and antioxidant properties (Ibrahim & Alqurashi, 2022; Pobiega et al., 2019). The type of solvent used to prepare propolis extract and the content of phenolic and flavonoid compounds have an important effect on its antimicrobial and antioxidant properties (Przybyłek & Karpiński, 2019).

Ethanolic extract of propolis has been used in fruit juices as a natural antifungal agent (Pobiega et al., 2019). Utilizing 2% of propolis extract in beef patties reduced microbial growth and lipid oxidation during storage (Vargas‐Sánchez et al., 2014). It has been reported that aqueous (4, 8, 12% v/w) and hydroalcoholic (0.4% v/w) extracts of propolis as natural preservatives extended the shelf life of chicken kebab (Mahdavi‐Roshan et al., 2022) and salami (Mafra et al., 2022), respectively. According to Vasilaki et al. (2019), adding the extract of propolis (300 mg/g drink) in the noncarbonated orange soft drink showed significant antimicrobial activity and increased the antioxidant activity and total phenolic content compared to the drink containing potassium sorbate as a preservative. Since propolis is classified in the GRAS list (Cottica et al., 2015; Santos et al., 2020) and is a nontoxic compound (Jansen‐Alves et al., 2019; Pobiega et al., 2019; Yang et al., 2017), it has the potential to be used as a natural antimicrobial and antioxidant preservative. This compound can be used for the improvement of the nutritional value of food products. The purpose of this research is to evaluate the effect of the ethanolic extract of propolis as a natural additive on the physicochemical, textural, microbial, sensory, and antioxidant properties of toast bread.

## 2 MATERIALS AND METHODS

### 2.1 Materials

The ethanolic extract of propolis was supplied by the Iranian Institute of medicinal plants. Wheat flour (82% extraction rate), sugar, milk, baker's yeast, salt, and vegetable oil as bread ingredients were purchased from a local store. The improving agent (DATEM) was purchased from Sahar Co. (Iran, Tehran). All the chemicals (Merck Darmstadt, Germany) were of analytical grade. DPPH was supplied by Sigma‐Aldrich (USA).

### 2.2 Propolis extract characterization

Evaluation of fat, protein, and ash content of propolis extract was carried out by Soxhlet (AOAC.935.38), Kjeldahl (AOAC 950.36), and gravimetric method, respectively (AOAC, 2006). The phenolic content was determined according to Kaur et al. (2008) with some modifications. First, 100 μL of propolis extract was mixed with 900 μL of distilled water and 1.5 mL of Folin–Ciocalteu reagent and the mixture was allowed to rest in the dark for 5 min at room temperature. Then, 2 mL of sodium carbonate 7.5% (w/v) was added, and the mixture was incubated in the dark for 90 min. The absorption was measured at 750 nm with an Ultraviolet–Visible spectrophotometer (SU‐6100‐Philler Scientific, USA). The results were expressed as mg GAE/g extract.

The antioxidant activity was evaluated by DPPH free radical scavenging method (Shehata et al., 2020). First, 0.5 mL of propolis extract was mixed with 3.5 mL of 0.1 mM DPPH methanolic solution. After half an hour of incubation in the dark at room temperature, the absorbance at 517 nm was measured by an Ultraviolet–Visible spectrophotometer (SU‐6100‐ Philler Scientific, USA).

### 2.3 Production of toast bread

Briefly, wheat flour and powdered ingredients were mixed with a dough mixer (Table 1). Then, propolis extract was added (0.1, 0.3, and 0.5%), as well as vegetable oil. The control contained no propolis extract. The dough mass was kept for 10 min in a proofer (Morshed Gohar, Iran) at 35°C and relative humidity of 88%. After dividing the dough into pieces of about 650 g, one‐third of the toast molds were filled with dough. The final proof was carried out in the proofer for 30 min. Afterward, the bread was baked in a rotary oven at 215°C for 10 min. After cooling, the bread samples were packed in polyethylene bags and stored at room temperature (Mohammadi Golchin et al., 2021).

**TABLE 1:** The ingredients used in toast bread formulation.

Ingredients	Percentage (%flour weight)
Wheat flour	100
Sugar	1.44
Improving agent	1.12
Bakery Yeast	3.2
Salt	1.42
Vegetable oil	2.88
Propolis extract	0.1–0.5

### 2.4 Analyses of toast bread

#### 2.4.1 Physicochemical tests

Moisture and ash contents were measured by the gravimetric method according to AOAC 925.10 and AOAC 923.03 methods, respectively (AOAC, 2006). The pH of breads was determined using a 7COMPACT‐METTLER Swiss pH meter, based on AACC 02‐52.01, and the specific volume was assessed via the rapeseed displacement method AACC10‐05.01 (AACC, 2000). All measurements were performed in triplicate.

#### 2.4.2 Color analysis

The effect of propolis extract on the color of bread was evaluated by the ColorFlex Hunter colorimeter. The color indices including L* (0 = black, 100 = white), a* (+: red; −: green), and b* (+: yellow; −: blue) were measured in triplicate. The total color difference with control (Δ*E*), chroma (color intensity), and browning index (BI) were calculated using Equations (1), (2), (3)), respectively (Çakmakçı et al., 2015; Mikulec et al., 2019).

(1) $$∆E=\sqrt{{\left(\mathrm{\Delta L}\right)}^{2}+{\left(\mathrm{\Delta a}\right)}^{2}+{\left(\mathrm{\Delta b}\right)}^{2}}$$

where Δ*L*, Δ*a*, and Δ*b* are the differences in lightness, redness, and yellowness, respectively.

(2) $$C^{*}=\sqrt{a^{*2}+b^{*2}}$$

(3) $$\mathrm{BI}=\left[100\left(X-0.31\right)\right]/0.17$$

where *x* = (a* + 1.75 L*)/(5.645 L* + a*–3.012b*).

#### 2.4.3 Texture analysis

The texture profile analysis of bread was carried out using a texture analyzer (CT3 10 K – Brookfield, USA) at a speed of 1 mm/s and 40% compression. The hardness, chewiness, cohesiveness, and fracturability of bread cubes (2 × 2 × 2 cm) were measured on the first, third, and fifth days of storage on six repetitions (Bourekoua et al., 2018).

#### 2.4.4 Total phenolic content and antioxidant activity

The dried bread sample (3 g) was mixed with 30 mL of ethanol (80% v/v) by a magnetic stirrer (Sci Finetech, South Korea) for 30 min. The mixture was centrifuged at 1792 *g* for 15 min (Pars Azma Iran). The supernatant was used to determine the phenolic content and antioxidant activity (three replicates for each sample). Five milliliters of supernatant was mixed with 20 mL of hexane, and after 20 min of centrifugation at 1792 *g*, the fat was separated by a syringe. The total phenolic content was measured using the Folin–Ciocalteu reagent (Bourekoua et al., 2018). One milliliter of Folin–Ciocalteu reagent was mixed with 1 mL of defatted supernatant and 10 mL of distilled water on a magnetic stirrer for 5 min. After adding 2 mL of sodium carbonate 7.5% (w/v) and incubating the mixture in the dark for 60 min, the absorbance of the sample was recorded at 750 nm using an Ultraviolet–Visible spectrophotometer (SU‐6100‐Philler Scientific, USA). The results were expressed as mg GAE/g.d.m.

For the evaluation of antioxidant activity, the free radical scavenging activity was evaluated using the DPPH method in triplicate (Piechowiak et al., 2020). One milliliter of the extracted solution and 5 mL of 0.1 mM DPPH solution were mixed. After incubating the mixture in the dark for 50 min, the absorbance was assessed at a wavelength of 517 nm by an Ultraviolet–Visible spectrophotometer (SU‐6100; Philler Scientific, USA).

#### 2.4.5 Molds enumeration

The mold count was performed according to ISO 21527‐2 (2008) on days 1, 3, and 5 after baking in triplicate. The serial dilutions were prepared for each sample and 0.1 mL of the corresponding dilution was spread in YGC agar medium (Merk, Germany) with a sterile spreader. The Petri dishes were incubated at 25°C for 3 days and the mold counts were expressed as the log CFU/g.

#### 2.4.6 Sensory analysis

The 9‐point hedonic scale was used for sensory analysis. Bread slices (2 × 2 × 2 cm) with a three‐digit code were randomly prepared for the 22 semitrained panelists. The evaluation was carried out for color, taste, texture, odor, aftertaste, and overall acceptance from 1 (strong dislike) to 9 (extremely like) scores (Mikulec et al., 2020).

#### 2.4.7 Statistic analysis

The significant difference between the data was determined by a one‐way analysis of variance in MINITAB16 software at a significance level of 95%. Tukey's and Fisher's tests were performed to compare the means.

## 3 RESULTS AND DISCUSSION

### 3.1 Characterization of ethanolic propolis extract

The results of the analysis of ethanolic propolis extract are presented in Table 2. The amounts of fat, protein, and ash were lower than propolis obtained from six geographical regions reported by Shehata et al. (2020), which were in the range of 8.64–15.60%, 1.83–2.89%, and 0.85–1.71%, respectively. The total phenolic content (293.72 mg GAE/L) was higher than those stated by Ulloa et al. (2017). The antioxidant activity (47.18%) was higher than water extracts of propolis obtained by Mahdavi‐Roshan et al. (2022) (15.83%) and lower than the ethanolic propolis extract reported by Ibrahim and Alqurashi (2022) (94.45%). Previous studies have reported that Iranian propolis extracts as a natural source of various antioxidants (Mahdavi‐Roshan et al., 2022; Mohammadzadeh et al., 2007; Najafpour Darzi et al., 2019). The origin of propolis, the extraction method, and the type of solvent used for extraction influence propolis extract characterization (Mafra et al., 2022).

**TABLE 2:** Fat, protein, ash, phenolic content, and antioxidant activity of ethanolic propolis extract.

Material	Fat (%)	Protein (%)	Ash (%)	Phenolic content (mgGAE/L)	Antioxidant activity (%)
Ethanolic extract of propolis	1.71	0.56	1.50	293.72	47.18

### 3.2 Physicochemical characteristics of bread

The results of the physicochemical properties of bread are presented in Table 3. No significant difference was observed in moisture content (*p* > .05). It seems that this is due to the use of low propolis concentrations in the present study. There was an increase in ash content with elevating propolis concentration (*p* < .05). The ash content of propolis samples collected from different regions of Morocco has been reported between 0.5 and 0.72% (El Menyiy et al., 2021) and calcium was the most important mineral found, followed by sodium, potassium, and magnesium (El Menyiy et al., 2021). Since the pH of propolis used was almost close to bread, the effect of propolis on pH value was not significant (*p* > .05). The specific volume of bread was not affected by propolis concentrations. Indeed, due to utilizing the low concentration of propolis, the interaction between the propolis and the gluten network did not affect the gas retention capacity inside the gluten network.

**TABLE 3:** The effect of ethanolic propolis extract on physicochemical properties of breads.

Sample	Moisture (%)	Ash (%)	pH	Specific volume (cm^3^/g)
EPE 0%	40.57 ± 2.09^a^	0.65 ± 0.04^b^	5.15 ± 0.14^a^	3.00 ± 0.36^a^
EPE 0.1%	41.33 ± 1.89^a^	0.69 ± 0.02^ab^	5.22 ± 0.17^a^	3.00 ± 0.23^a^
EPE 0.3%	41.12 ± 0.09^a^	0.71 ± 0.02^a^	5.22 ± 0.27^a^	2.50 ± 0.07^a^
EPE 0.5%	41.90 ± 0.10^a^	0.71 ± 0.02^a^	5.19 ± 0.21^a^	2.79 ± 0.38^a^

*Note*: Results are presented as a mean value ± SD (*n* = 3), EPE 0%: bread without ethanolic propolis extract (Control), EPE 0.1%: bread containing 0.1% ethanolic propolis extract, EPE 0.3%: bread containing 0.3% ethanolic propolis extract, and EPE 0.5%: bread containing 0.5% ethanolic propolis extract. The different superscript letters are significant in the same column (*p* < .05).

### 3.3 Texture analysis

An increase in the content of propolis significantly (*p* < .05) affected the hardness of bread samples (Table 4). The hardness of bread with 0.5% propolis extract was about 1 N more compared to the control on the fifth day. In addition, the hardness increased from day 1 to 5. The results of chewability and fracturability were also in agreement with hardness. Probably, the presence of propolis interferes with and intensifies the formation of amylopectin crystals in starch and leads to an increase in the crystalline‐to‐amorphous starch ratio. However, proving this claim requires additional analysis such as ATR‐FTIR and XRD.

**TABLE 4:** Texture evaluation of breads with ethanolic propolis extract during storage.

Sample	Day	Hardness (*N*)	Chewiness (mJ)	Cohesiveness	Fracturability (*N*)
EPE 0%	1	0.70 ± 0.15^e^	1.54 ± 0.74^e^	0.44 ± 0.10^cd^	0.35 ± 0.32^f^
EPE 0%	3	1.11 ± 0.42^bcde^	4.09 ± 1.17^cd^	0.48 ± 0.04^bc^	0.93 ± 0.58^de^
EPE 0%	5	1.21 ± 0.51^bc^	5.31 ± 2.91^c^	0.49 ± 0.05^bc^	1.28 ± 0.36^cd^
EPE 0.1%	1	0.75 ± 0.16^de^	1.88 ± 0.82^de^	0.6 ± 0.06^a^	0.35 ± 0.24^f^
EPE 0.1%	3	1.11 ± 0.40^bcd^	4.56 ± 2.20^c^	0.53 ± 0.03^ab^	1.02 ± 0.42^de^
EPE 0.1%	5	1.29 ± 0.43^b^	6.28 ± 2.63^bc^	0.5 ± 0.06^bc^	1.32 ± 0.73^cd^
EPE 0.3%	1	0.87 ± 0.33^cde^	2.11 ± 1.27^c^	0.39 ± 0.08^d^	0.64 ± 0.59^ef^
EPE 0.3%	3	1.26 ± 0.42^bc^	4.98 ± 2.05^c^	0.43 ± 0.06^cd^	1.79 ± 0.97^bc^
EPE 0.3%	5	2.55 ± 0.61^a^	8.75 ± 3.35^a^	0.50 ± 0.05^bc^	2.60 ± 0.67^a^
EPE 0.5%	1	0.74 ± 0.18^de^	1.88 ± 0.87^de^	0.47 ± 0.06^bc^	0.42 ± 0.32^ef^
EPE 0.5%	3	1.28 ± 0.33^b^	4.63 ± 1.46^c^	0.54 ± 0.05^ab^	1.25 ± 0.24^cd^
EPE 0.5%	5	2.19 ± 0.53^a^	8.18 ± 3.23^ab^	0.61 ± 0.14^a^	2.27 ± 0.80^ab^

*Note*: Results are presented as a mean value ± SD (*n* = 6), PE 0%: bread without propolis extract (Control), PE 0.1%: bread containing 0.1% propolis extract, PE 0.3%: bread containing 0.3% propolis extract, and PE 0.5%: bread containing 0.5% propolis extract. The different superscript letters are significant in the same column (*p* < .05).

The cohesiveness of bread was not affected by propolis. Therefore, the differences observed were more related to the structure and the porous texture of the bread than to propolis.

### 3.4 Color

The color analysis results are shown in Table 5. The L* value (lightness) decreased significantly with the increase of ethanolic propolis extract in bread (*p* < .05). The increase of the propolis level up to 0.5% caused a significant increase in color intensity and browning index compared to the control. According to Cottica et al. (2015), the ethanolic extract of propolis leads to more color changes than the aqueous extract due to the more easily extraction of darker pigments by ethanol than water. In addition, the thermal process also induces the polymerization of phenolic compounds and creates darker compounds (Cottica et al., 2015).

**TABLE 5:** Changes in color values of breads with ethanolic propolis extract.

Sample	L*	a*	b*	Chroma	BI	Δ*E*
EPE 0%	53.80 ± 0.91^a^	−0.86 ± 0.44^a^	32.05 ± 0.48^b^	32.07 ± 0.47^b^	83.72 ± 3.24^b^	–
EPE 0.1%	50.75 ± 1.16^b^	−0.52 ± 0.05^a^	31.62 ± 0.36^b^	31.62 ± 0.37^b^	90.05 ± 3.54^ab^	3.13 ± 1.12^a^
EPE 0.3%	51.09 ± 1.01^b^	−1.73 ± 0.49^b^	32.34 ± 0.34^ab^	32.39 ± 0.36^ab^	90.26 ± 3.58^ab^	2.90 ± 1.06^a^
EPE 0.5%	51.12 ± 0.30^b^	−1.87 ± 0.42^b^	33.17 ± 0.45^a^	33.22 ± 0.47^a^	93.50 ± 2.56^a^	3.11 ± 0.47^a^

*Note*: Results are presented as a mean value ± SD (*n* = 3), EPE 0%: bread without ethanolic propolis extract (Control), EPE 0.1%: bread containing 0.1% ethanolic propolis extract, EPE 0.3%: bread containing 0.3% ethanolic propolis extract, and EPE 0.5%: bread containing 0.5% ethanolic propolis extract. The different superscript letters are significant in the same column (*p* < .05).

Propolis decreased and increased a* and b* values in bread, respectively. The color of propolis strongly depends on the plant's origin and varies from yellow to brown and black (Cottica et al., 2015; Jansen‐Alves et al., 2019). The brown nature of propolis besides the presence of yellowish flavonoids resulted in an increase of b* in bread samples. This is in line with the study of Jansen‐Alves et al. (2019). According to their report, the b* value increased in cake by using the ethanolic propolis extract. It should be noted that the color difference (ΔE) of bread containing propolis was not affected by the concentrations of propolis extract used in the present study.

### 3.5 Total phenolic content (TPC) and antioxidant activity

**FIGURE 1:**
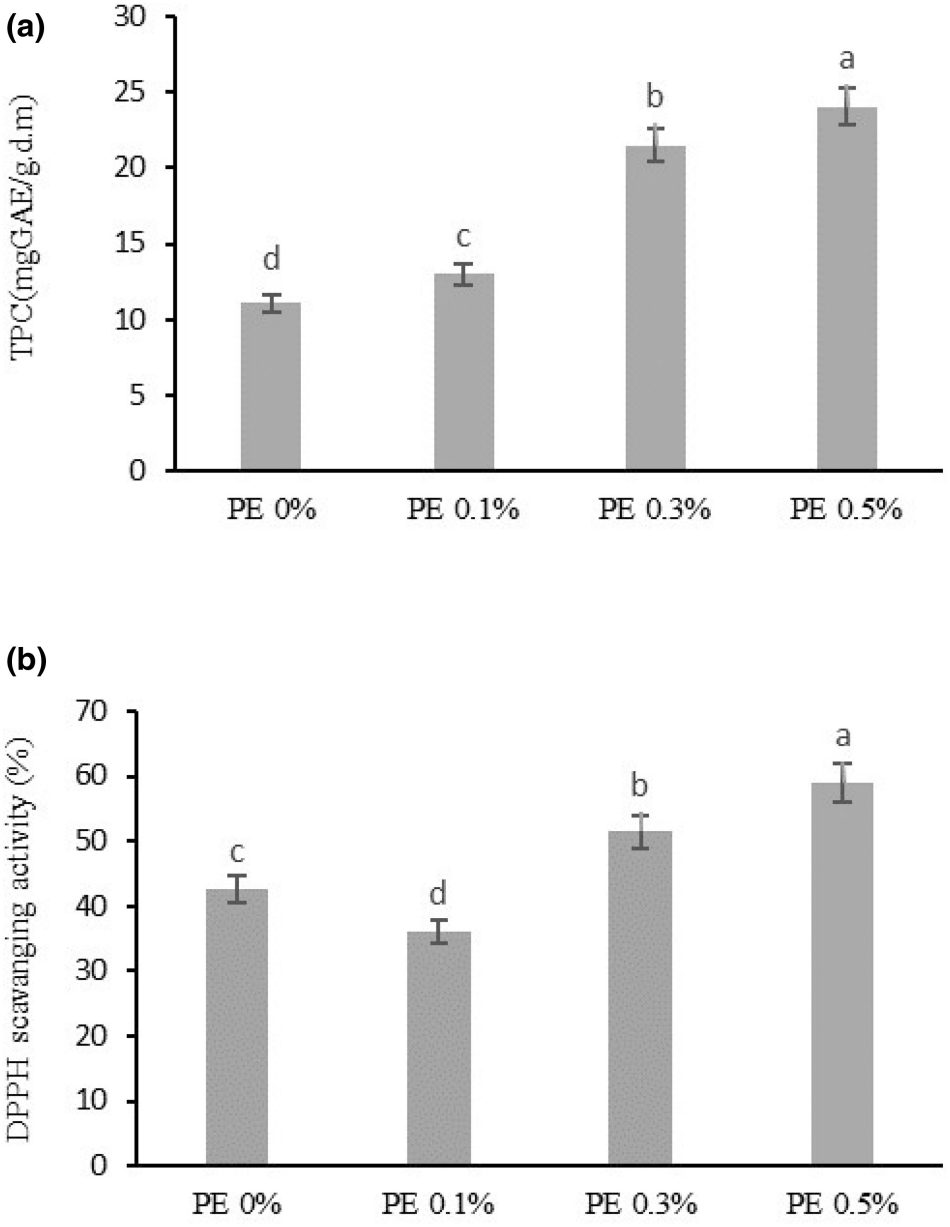
Results of total phenolic content (a) and antioxidant activity (b) of breads containing ethanolic propolis extract. EPE 0%: bread without ethanolic propolis extract (Control), EPE 0.1%: bread containing 0.1% ethanolic propolis extract, EPE 0.3%: bread containing 0.3% ethanolic propolis extract, and EPE 0.5%: bread containing 0.5% ethanolic propolis extract. The different superscript letters are significant (*p* < .05).

The total phenolic content of bread samples increased with the addition of propolis (*p* < .05) from 11.06 mgGAE/g in control to 24.02 mgGAE/g in the bread containing 0.5% propolis (Figure 1a). The propolis extract contains phenolic compounds such as caffeic acid, coumaric acid, ferulic acid, kaempferol, quercetin, chlorogenic acid, cinnamic acid, and syringic acid (Ibrahim & Alqurashi, 2022; Przybyłek & Karpiński, 2019). The previous studies demonstrated that propolis extract increased the total phenolic content in cake (Jansen‐Alves et al., 2019) and beverages (Vasilaki et al., 2019). Using propolis at a level of 0.5% increased the antioxidant activity (DPPH free radical scavenging capacity) of bread (59.03%) compared to the control (42.59%) (Figure 1b). The antioxidant activity of the ethanolic extract of propolis in the present study was 47.18% (Table 2). The antioxidant properties of propolis are due to the presence of flavonoid and phenolic compounds which are different according to the type of plant origin, and geographical regions (Irigoiti et al., 2021). Cottica et al. (2015) and Jansen‐Alves et al. (2019) also reported higher antioxidant activity by the addition of propolis extracts in dairy beverages (8, 40, and 80 ppm) and cake (0.1%), respectively.

### 3.6 Mold growth

The results of the mold enumeration are presented in Table 6. The effect of propolis extract showed no significant difference on day 1. The mold counts were significantly lower in bread samples containing 0.3 and 0.5% propolis extract on the third and fifth days. The lowest mold count was observed in bread containing 0.5% propolis extract. Despite the increase in mold growth during the storage period, the mold count in the bread with 0.5% propolis extract was not significantly different on the fifth and first days of storage (*p* > .05), probably due to the appropriate antifungal activity of propolis. A decrease in mold growth by adding ethanolic extracts of propolis at concentrations of 0.5, 1, and 1.5% in thyme labneh after 21 days of storage (Ibrahim & Alqurashi, 2022), an increase in the shelf life of sausage from 12 to 21 days by using 0.6% propolis ethanolic extract (Ali et al., 2010), and prevention from the growth of *Penicillium expansum* and synthesis of patulin in an apple juice containing 0.2% ethanolic extract of propolis (Silici & Karaman, 2014) have been reported previously. There is also a report about the inhibitory effect of the water extract of propolis on mold growth in fresh shibuta fillet during storage (Duman & Özpolat, 2015).

**TABLE 6:** The effect of ethanolic propolis extract on mold counts (log CFU/g) of breads during storage.

Sample	Day of storage		
	1	3	5
EPE 0%	0.00 ± 0.00^aC^	2.33 ± 0.04^aB^	3.13 ± 0.04^aA^
EPE 0.1%	0.00 ± 0.00^aC^	2.19 ± 0.07^aB^	2.94 ± 0.05^aA^
EPE 0.3%	0.00 ± 0.00^aB^	1.02 ± 0.89^bAB^	1.99 ± 0.11^bA^
EPE 0.5%	0.00 ± 0.00^aA^	0.00 ± 0.00^cA^	0.53 ± 0.92^cA^

*Note*: Results are presented as a mean value ± SD (*n* = 3), EPE 0%: bread without ethanolic propolis extract (Control), EPE 0.1%: bread containing 0.1% ethanolic propolis extract, EPE 0.3%: bread containing 0.3% ethanolic propolis extract, and EPE 0.5%: bread containing 0.5% ethanolic propolis extract. The different superscript lowercase and uppercase letters are significant (*p* < .05) in each identical column and row, respectively.

The antimicrobial properties of propolis are related to its bioactive compounds which mainly include flavonoids, phenolic acids, aldehydes, and ketones (Irigoiti et al., 2021). In addition, a synergistic effect between propolis phenolic compounds and Maillard reaction products may be observed, as a synergistic effect has been observed for the antifungal activity of plant extracts in bread (Axel et al., 2017).

The a_w_ of bread is between 0.94 and 0.97, which makes it susceptible to mold growth. There is a major concern for the bakery industry and consumers about the growth of molds and the resulting waste problem. Also, the presence of mycotoxins due to mold contamination in cereal products is a crucial issue related to consumer health. Nowadays, consumers' demand is more for chemical additive‐free bakery products. Besides, it seems to be necessary to use natural preservatives due to the resistance of several fungi to chemical preservatives. Examples of some natural preservatives used in bread are lactic acid bacteria (LAB) in sourdough, yeasts other than baker's yeast along with LAB strains or without them, antifungal peptides, and plant extracts (Axel et al., 2017). According to the results of this research, propolis has the potential to be used as a natural preservative in toast bread.

### 3.7 Sensory analysis

**FIGURE 2:**
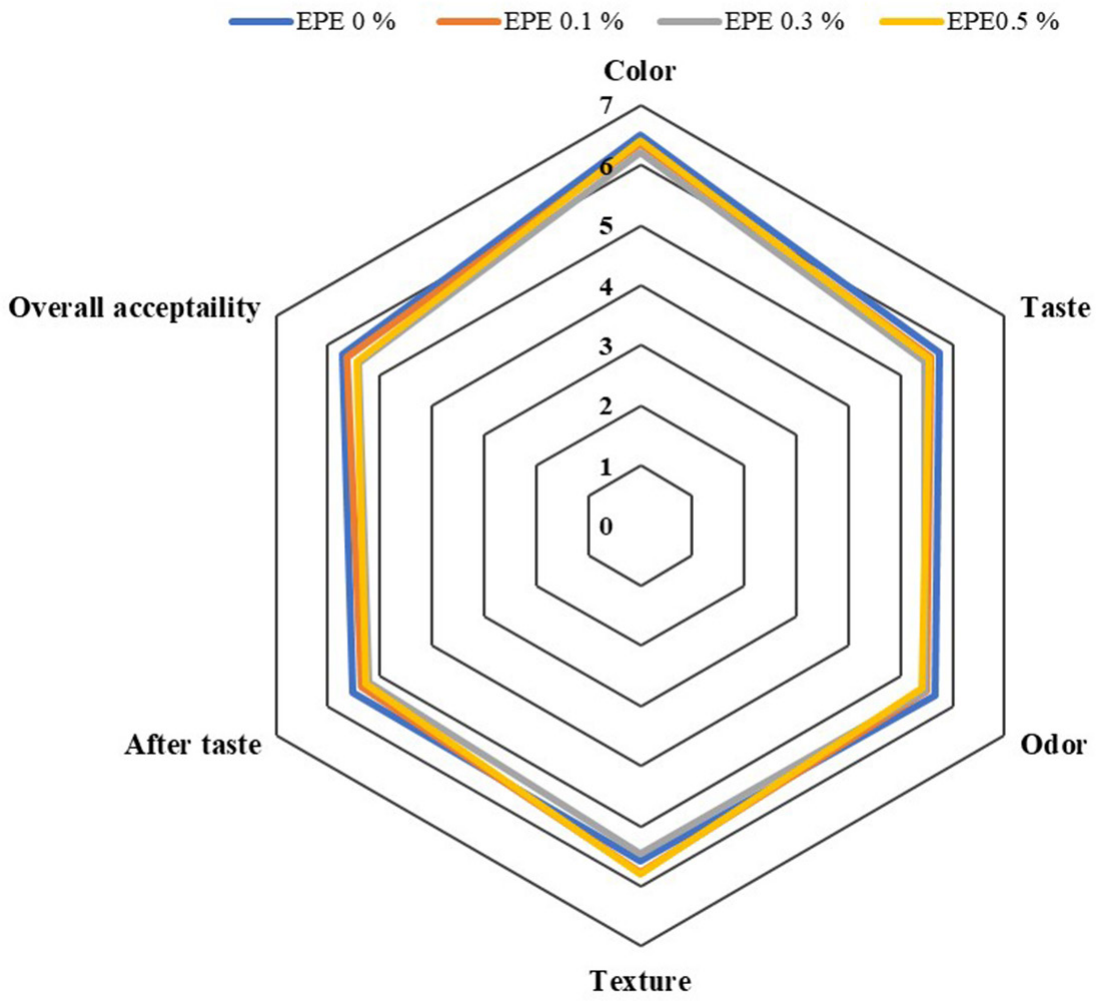
Sensorial values of breads containing ethanolic propolis extract. EPE 0%: bread without ethanolic propolis extract (Control), EPE 0.1%: bread containing 0.1% ethanolic propolis extract, EPE 0.3%: bread containing 0.3% ethanolic propolis extract, and EPE 0.5%: bread containing 0.5% ethanolic propolis extract.

The results of the sensory analysis are shown in Figure 2. There are slight differences in most sensory indices which is probably due to the use of low concentration of propolis extract in breads. The color scores in sensory evaluation are in line with the results of ∆E obtained in color analysis. Since the ∆*E* ranges were between 1 and 3 and in some cases slightly higher than 3, in the color evaluation of samples by panelists, the color difference was not distinguishable (Mikulec et al., 2019).

Propolis has a strong and distinct odor, and adding it to food can lead to an unpleasant odor and color change (Santos et al., 2020) and its strong taste and odor remains in the product (Jansen‐Alves et al., 2019). Preparing propolis extracts appropriately and using proper levels in a food formulation are significant challenges for the industry. The propolis utilization in food must be chosen in such a way that minimizes the unpleasant organoleptic properties while maintaining beneficial aspects for humans and the preservation of food (Pobiega et al., 2019). Addition of 0.5% or fewer amounts of propolis extract to several products such as fish (Duman & Özpolat, 2015), salami (Mafra et al., 2022), yogurt (Santos et al., 2020), and apple juice (Luis‐Villaroya et al., 2015) showed sensory satisfaction which is in line with the present study.

## 4 CONCLUSION

The results demonstrated that EPE was effective in the inhibition of mold growth of toast bread during storage and improved the antioxidant characteristics. The overall sensory acceptance of bread samples was acceptable. The function of EPE was dose dependent and the proper concentration used in toast bread was 0.5%. Therefore, EPE can be used as a valuable natural additive with antimicrobial and antioxidant functions in toast bread. Further research is needed to replace propolis extract with chemical preservatives in bread.

## AUTHOR CONTRIBUTIONS

**Saba Hosseini Khabbazi:** Funding acquisition (lead); investigation (equal); methodology (equal); resources (equal); writing – review and editing (equal). **Samar Mansouripour:** Conceptualization (equal); data curation (equal); formal analysis (equal); project administration (equal); software (lead); supervision (equal); validation (equal); writing – original draft (lead); writing – review and editing (lead). **Solmaz Saremnezhad:** Conceptualization (equal); data curation (equal); formal analysis (equal); methodology (equal); project administration (equal); supervision (equal); validation (equal); writing – review and editing (equal).

## FUNDING INFORMATION

This research received no specific grant from any funding source.

## CONFLICT OF INTEREST STATEMENT

The authors declare that there is no conflict of interest in this study.

## ETHICS STATEMENT

This study does not involve any human or animal testing.

## INFORMED CONSENT

Written informed consent was obtained from all study participants.

## Data Availability

The data that support the findings of this study are available from the corresponding author upon reasonable request.
